# Subtitle Engagement Varies with Audio–Subtitle Language–Script Pairing: Evidence from Hindi–English Bilinguals with an English-Medium Instruction Background

**DOI:** 10.3390/vision10020036

**Published:** 2026-06-22

**Authors:** Inka Romero-Ortells, Manuel Perea, Eva Gutierrez-Sigut, Jon Andoni Duñabeitia

**Affiliations:** 1 Centro de Investigación Nebrija en Cognición (CINC), Universidad Nebrija, 28043 Madrid, Spain; iromeroortells@nebrija.es (I.R.-O.); jdunabeitia@nebrija.es (J.A.D.); 2 Departament de Metodologia de les Ciències del Comportament and ERI Lectura, Universitat de València, 46010 Valencia, Spain; 3 Department of Psychology, University of Essex, Colchester CO4 3SQ, UK; eva.gutierrez@essex.ac.uk; 4 DCAL Research Centre, University College London, London WC1E 6BT, UK

**Keywords:** bilingualism, English-medium instruction, eye-tracking, instructional video, language background, subtitling

## Abstract

Subtitles often attract visual attention even when they are not necessary for comprehension. In the present eye-tracking experiment, we examined whether attention to subtitles in instructional videos varies as a function of audio–subtitle language–script pairing in Hindi–English bilinguals with an English-medium instruction (EMI) background. Native Hindi participants viewed videos in three conditions: English audio with English subtitles (L2–L2), Hindi audio with Hindi subtitles (L1–L1), and English audio with Hindi subtitles (L2–L1). In the L2–L2 condition, gaze was distributed similarly across speakers’ faces and subtitles. In contrast, in both Hindi-subtitle formats, viewers allocated more dwell time to the speakers’ faces than to the subtitles. Comprehension scores did not differ significantly across conditions. These findings suggest that subtitle engagement among EMI bilinguals is not solely determined by the presence of subtitles but is also modulated by the properties and perceived utility of the written channel. More generally, our results caution against the view that subtitle engagement is uniformly automatic across multilingual instructional settings.

## 1. Introduction

Instructional videos require viewers to coordinate speech with dynamic visual information and, in some cases, on-screen text. Subtitles are especially relevant in multimodal environments because they recode transient speech into a written format that can guide attention during viewing [1]. For simplicity, we use the term “subtitles” throughout to refer to all on-screen text while recognising that L2–L2 and L1–L1 (same-language) conditions correspond more strictly to captions. Previous eye-tracking experiments have shown that viewers often allocate substantial visual attention to subtitles even when the audio is fully intelligible [2,3,4]. For example, d’Ydewalle et al. (1991) reported that viewers spent approximately 30% of their viewing time looking at the subtitles even when the soundtrack was in their native language [4]. This pattern has often been interpreted as evidence that subtitles can attract attention in a relatively automatic way, even when they are not strictly necessary for comprehension; for convenience, we refer to this view as a strong automatic attention account. In educational environments, same-language subtitles have been argued to support reading development by presenting spoken and written input together during video viewing [5]. Evidence from India suggests that same-language subtitles can encourage incidental reading and improve word recognition, particularly among viewers with some functional reading ability [6,7].

However, whether this tendency generalises across bilingual populations, literacy profiles, and writing systems remains unresolved. For example, recent eye-tracking research has shown that subtitle engagement is not uniform across readers. Lopukhina et al. (2025, 2026) reported that children with emerging decoding skills engaged only minimally with same-language subtitles, whereas more proficient readers showed more systematic, although still incomplete, attention to subtitle text [8,9]. These findings suggest that subtitle availability on screen alone does not guarantee visual attention allocation; rather, engagement with subtitles might depend on experience with the subtitle language. In this context, it is important to specify when subtitles attract attention and for whom.

Subtitle engagement can vary with language proficiency and processing demands. When the audio is in an L2, same-language subtitles can support speech segmentation and lexical access, whereas L1 subtitles have been shown to facilitate general comprehension and reduce subjective effort [10,11]. However, providing both written and spoken information does not mean viewers will rely on them equally. Instead, attention allocation is likely to depend on which information source is easier to process and more useful for comprehension at a given moment. In line with the Cognitive Theory of Multimedia Learning [12,13], learners do not process all available information uniformly; rather, they allocate attention to reduce unnecessary processing demands and prioritise information relevant to the task [14]. For bilinguals with a background in English-medium instruction (EMI)—where English is the primary language of formal schooling, even though it is not the home language [15]—differences in perceived accessibility are often related to the degree of practice in reading English. This extensive practice may make English text comparatively efficient to process during academic tasks, even when English is formally an L2. From a multimedia-learning perspective, this efficiency should matter because learners may allocate attention to information sources that are likely to support comprehension relative to their processing costs [12,13,14].

Consistent with this view, Negi and Mitra [16] reported that Marathi/English bilinguals showed higher instructional efficiency and lower cognitive load (measured via EEG and self-reports) when viewing English videos with L1 (Marathi) subtitles rather than L2 (English) subtitles. The eye-tracking data indicated that viewers looked at L1 subtitles more consistently across the video, whereas the viewing of L2 subtitles decreased over time, suggesting that L1 subtitles provided more useful written support for comprehension in that context. Furthermore, Wang and Pellicer-Sánchez (2023) found that even when both L1 (Chinese) and L2 (English) subtitles were presented simultaneously, Chinese/English bilinguals allocated more visual attention to L1 translations than to L2 target words, even though attention to the English items was the best predictor of vocabulary learning [17]. This pattern is also consistent with the general idea of an eye–ear relationship in subtitle viewing, whereby auditory language processing can shape when and how viewers allocate their attention to subtitles [18]. However, this relation may not be fixed, as the written stream may be useful depending on the language, script, and literacy profile of the viewer. Taken together, these findings favour a literacy utility account of subtitle engagement. On this view, gaze allocation reflects the expected benefit of the written channel relative to the processing demands imposed by the auditory stream, the visual scene, and the subtitle script.

The present study focuses on Hindi–English bilinguals who completed their education through English-medium instruction (EMI) in India and were studying in the United Kingdom at the time of testing. This population is theoretically informative because linguistic nativeness and academic literacy dominance may not coincide [19]. Although Hindi is participants’ first language, English has been the dominant language of their formal education. In India, English occupies a privileged position in education and higher education, which may confer a disproportionate role in formal literacy practices relative to other languages [20]. Accordingly, English functions for many as the more practised language for academic reading, despite its L2 status [21]. This is consistent with the “double divide” in Indian sociolinguistics, where English is positioned as the primary language of instrumental and academic power, while regional languages like Hindi, despite their integrative value, are often sidelined in formal literacy practices [22]. This possibility matters for eye-movement behaviour because reading efficiency depends not only on language knowledge but also on the frequency and context of subtitle language use.

Furthermore, Devanagari, the script used to write Hindi (e.g., देवनागरी), and the Latin alphabet differ in their orthographic structure. Whereas Latin script is linearly arranged, Devanagari includes vowel signs, or *matras*, that attach above, below, or on either side of consonants [23]. Thus, Hindi–English bilinguals with an EMI background provide a compelling case study. Because EMI education serves as a proxy for extensive practice in English reading, this group allows us to investigate whether subtitle engagement is driven solely by a listener’s native language (linguistic nativeness) or modulated by instructional background and script-specific processing demands. Importantly, this sociolinguistic configuration is not unique to India; across many EMI settings worldwide, English occupies a privileged role in higher education, while local languages remain central to everyday life [15].

In the present experiment, we examined how attention is distributed across three audio–subtitle combinations to contrast three competing accounts of subtitle engagement. Under a strong automatic attention account, subtitles should attract gaze whenever they are present and legible; thus, dwell time on subtitles should remain substantial and relatively comparable across the L2–L2 and L1–L1 conditions [2,3,4,24]. Alternatively, a linguistic nativeness account would predict that viewers allocate more attention to subtitles in their first language (Hindi, L1) to maximise comprehension, especially when processing an L2 audio stream [18]. Finally, under a literacy utility framework, subtitle engagement is expected to vary based on the perceived task utility and long-term script familiarity associated with a viewer’s formal instruction history rather than linguistic nativeness alone [8,9]. For Hindi–English EMI bilinguals, years of English-medium instruction may have created a literacy asymmetry in which English is the most practised script for academic reading, while Hindi may be less entrenched in formal literacy practices [19,20,21,22]. Based on previous evidence that subtitle engagement depends on reading skill, literacy experience, and the processing demands of the written channel [5,6,7,8,9], we predicted greater engagement in the L2–L2 condition than in the two Hindi-subtitle conditions.

## 2. Materials and Methods

### 2.1. Participants

Thirty native speakers of Hindi (15 women; age range = 21–37 years, M = 26.07, SD = 4.06) from the University of Essex (Colchester, UK) took part in the experiment. All reported an English-medium educational background from kindergarten onward. We selected this cohort because long-term EMI provides a motivated, though indirect, proxy for sustained academic reading in English and for greater familiarity with English than with Hindi as a language of formal study. This approach recognises that, for many EMI learners, English functions as the language of schooling—the domain in which formal literacy is most extensively practised—whereas regional languages may receive less sustained use in high-level academic training [15].

An a priori power analysis conducted in G*Power (release 3.1.9.7, Düsseldorf, Germany) [25] indicated that a sample of 30 participants would provide approximately 90% power to detect a medium-sized within-subject interaction (*f* = 0.25) at *α* = 0.05, assuming a conservative correlation of 0.50 among repeated measures. All participants completed a brief survey on language background and audiovisual media habits. All reported regular engagement with audiovisual content. Participants also reported normal or corrected-to-normal vision and no history of language-related disorders. The University of Essex Ethics Committee approved this study (ETH2324-0585), and all participants provided informed consent and received course credit for participation.

### 2.2. Materials

**Figure 1:**
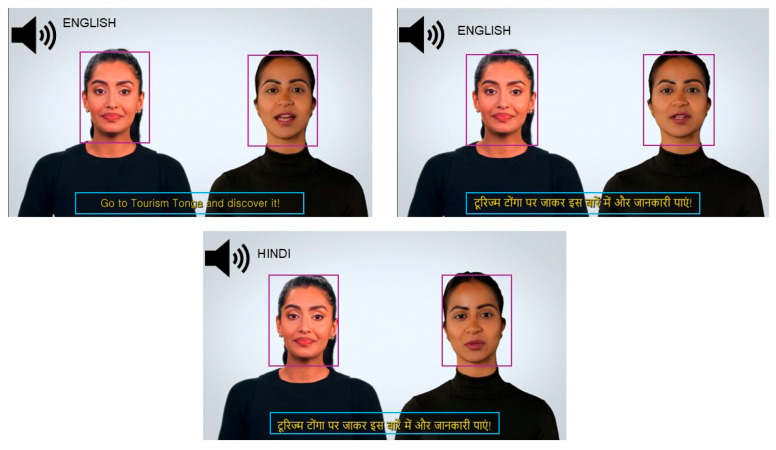
An example frame from the instructional videos showing the two avatars, the subtitle area, and the Areas of Interest (AOIs) used in the analyses (Face, Subtitles). The three panels represent the structural configurations across the tested audiovisual conditions: L2–L2 (English audio/English subtitles), L2–L1 (English audio/Hindi subtitles), and L1–L1 (Hindi audio/Hindi subtitles).

The stimuli were three instructional videos (approximately 2 min 15 s each) about geographically diverse locations (Belize, Comoros, and Tonga), adapted into Hindi from materials developed by Romero-Ortells et al. (2026) [26]. The videos were created using Synthesia (2.0, Synthesia Ltd., London, UK) [27] and depicted two adult female avatars engaged in a conversational exchange. Using two speakers allowed us to examine the distribution of gaze across socially relevant facial information and the subtitle region (see Figure 1).

Each avatar had one British English voice track and one Hindi voice track; the track used depended on the condition. Avatar appearance, video length, and scene structure were held constant across versions to maximise comparability across conditions. We used a within-subject design with three audiovisual pairings: L2–L2 (English audio, English subtitles), L1–L1 (Hindi audio, Hindi subtitles), and L2–L1 (English audio, Hindi subtitles). The Hindi-audio/English-subtitle condition was not included because this study was designed to test three theoretically motivated pairings rather than a fully crossed 2 × 2 manipulation. Informal pilot feedback from native Hindi speakers further indicated that Hindi-mediated academic instruction supported by English text was uncommon for this population. Thus, the primary planned contrasts were L2–L2 versus L1–L1 and L2–L2 versus L2–L1; the L1–L1 versus L2–L1 comparison was included to test whether engagement with Hindi subtitles changed as a function of audio language.

Subtitles were added in Adobe Premiere Pro 2025 (version 25.6.4, CA, USA) [28]. We attempted to match subtitle presentation as closely as possible across languages at the file level, considering mean characters per second, mean words per minute, line breaks, and exposure duration, within the constraints imposed by differences between the Latin and Devanagari scripts. Subtitles were presented at the bottom centre of the screen in a sans-serif font (Roboto 48), with a maximum of 42 characters per line and exposure times of approximately 4–6 s [29,30]. All Hindi translations and subtitle files were checked by a professional translator.

The Supplementary materials are available on OSF at https://osf.io/69mys/overview?view_only=285dd5422bb94a148840870a8da7dbae (accessed on 15 April 2026).

### 2.3. Apparatus and Measures

Eye movements were recorded using an EyeLink^®^ 1000 Plus desktop-mounted eye tracker (SR Research Ltd., Ottawa, ON, Canada) sampling at 1000 Hz [31]. Stimuli were presented on a 24″ Asus monitor (1280 × 1024 resolution), and audio was delivered via high-definition headphones (Hercules HDP DJ60). Participants were seated 60 cm from the screen, using a chin-and-forehead rest to minimise head movement.

Rectangular Areas of Interest (AOIs) were defined to enclose each avatar’s face and the subtitle region at the bottom of the screen. For the inferential analyses, dwell time across the two face AOIs was summed to yield a single Face measure for each trial. Dwell time (cumulative fixation duration) was extracted for each AOI using EyeLink^®^ Data Viewer (SR Research Ltd., ON, Canada) [32], along with total valid sample time per trial (excluding blinks and track loss). Fixations were defined using the EyeLink^®^ default event parser.

### 2.4. Procedure

The experiment took place in a quiet laboratory room. After providing consent and receiving instructions, participants completed a 9-point calibration and validation procedure. Re-calibration was performed if the average validation error exceeded 0.5° or if the maximum error at any single point exceeded 1.0°. A fixation mark appeared at the location of the left speaker’s face as that speaker initiated the dialogue. The session began with a 30 s practice trial. A drift correction was performed prior to the start of each of the videos.

Each participant viewed three videos, experiencing each audiovisual condition once. Video–condition pairings were counterbalanced across participants using a Latin square design so that each video appeared equally often in each condition within the sample. Ten true/false comprehension questions followed each video to ensure participants’ sustained attention to the content; these scores were treated as a comprehension check rather than as a primary dependent variable. Questions were presented in English, matching the participants’ language of academic instruction.

### 2.5. Data Preparation and Analysis

For each trial, proportional dwell time was calculated by dividing AOI dwell time by total valid sample time, excluding blinks and periods of track loss. Fixations were defined using the EyeLink default event parser. No trials were removed because of excessive track loss. Each condition consisted of a single, extended video trial (2 min 15 s), an approach used in previous eye-tracking experiments with comparable video stimuli [17,26]. Given that each participant saw each condition only once, analyses were conducted at the trial level using proportional dwell times. Statistical analyses were conducted in JASP (0.95.4, JASP 2025) [33], using repeated-measures ANOVAs followed by Bonferroni-corrected pairwise comparisons for significant interactions.

## 3. Results

Invalid samples due to blinks and track loss were excluded from the eye movement analyses (L2–L2: 3.7%; L1–L1: 3.7%; L2–L1: 3.1%). Descriptive statistics on the proportional dwell times for faces and subtitles across conditions are presented in Table 1. To confirm that participants attended to the videos, we first analysed comprehension accuracy across conditions. Accuracy was similar in all three formats (L2–L2: *M* = 7.77, *SD* = 1.55; L2–L1: *M* = 7.50, *SD* = 1.72; L1–L1: *M* = 7.43, *SD* = 1.68; *F*(2, 58) = 0.71, *p* = 0.495, *η_p_*^2^ = 0.024).

We first examined whether subtitle engagement relative to the speakers’ faces differed in the two same-language conditions (L1–L1, L2–L2). To that end, we conducted a 2 (Condition: L1–L1, L2–L2) × 2 (AOI: Face vs. Subtitle) repeated-measures ANOVA on proportional dwell time. There were no significant main effects of Condition, *F*(1, 29) = 0.58, *p* = 0.452, *η_p_*^2^ = 0.020, or AOI, *F*(1, 29) = 2.63, *p* = 0.115, *η_p_*^2^ = 0.083. Critically, the Condition × AOI interaction was significant, *F*(1, 29) = 16.46, *p* < 0.001, *η_p_*^2^ = 0.362.

This interaction reflected that in the L1–L1 format, participants allocated significantly more dwell time to the speakers’ faces than to the subtitles, *t*(29) = 3.00, *p* = 0.033, whereas this difference did not appear in the L2–L2 condition, *t*(29) = −0.38, *p* = 1.000. This interaction also showed that subtitle dwell time was significantly greater in L2–L2 than in L1–L1, *t*(29) = −3.92, *p* = 0.003.

We next compared subtitle engagement when the video was in L2 (English) across L1 (Hindi) and L2 (English) subtitles. To that end, we used a 2 (Subtitle language: L1, L2) × 2 (AOI: Face, Subtitles) repeated-measures ANOVA on proportional dwell time. The main effect of AOI was significant, *F*(1, 29) = 5.81, *p* = 0.022, *η_p_*^2^ = 0.167, but the main effect of subtitle language was not, *F*(1, 29) = 0.75, *p* = 0.393, *η_p_*^2^ = 0.025. Notably, the Subtitle language × AOI interaction was significant, *F*(1, 29) = 41.72, *p* < 0.001, *η_p_*^2^ = 0.590. This interaction reflected that participants allocated more dwell time to the speakers’ faces than to the subtitles when the subtitles were in L1 (Hindi), *t*(29) = 4.93, *p* < 0.001, whereas viewing times for faces and subtitles did not differ when subtitles were in L2 (English), *t*(29) = −0.382, *p* = 1.000. In addition, the proportion of subtitle dwell time was also lower when subtitles were in L1 (Hindi) than L2 (English), *t*(29) = 6.47, *p* < 0.001.

Finally, we examined whether subtitle engagement, when the subtitles were in L1 (Hindi), depended on whether the audio was in L1 (Hindi) or L2 (English). To that end, we used a 2 (Audio language: L1, L2) × 2 (AOI: Face, Subtitles) repeated-measures ANOVA on proportional dwell time. The ANOVA revealed a significant main effect of AOI, *F*(1, 29) = 17.70, *p* < 0.001, *η_p_*^2^ = 0.379, indicating greater dwell time on faces (*M* = 0.701) than on subtitles (*M* = 0.275). We did not find a significant main effect of Audio Language, *F*(1, 29) = 1.07, *p* = 0.310, *η_p_*^2^ = 0.036, or a significant interaction between the two factors, *F*(1, 29) = 1.52, *p* = 0.227, *η_p_*^2^ = 0.050.

## 4. Discussion

The present study examined how Hindi–English bilinguals with an English-medium instruction background allocated visual attention across instructional videos with different audio–subtitle pairings. Our results showed a distinct shift in gaze behaviour based on the language–script pairing of the subtitles: participants distributed gaze similarly between faces and subtitles in the L2-audio/L2-subtitle condition (English audio with English subtitles) but spent more time on faces than on subtitles when subtitles were presented in Hindi (L1). This pattern is more consistent with a literacy utility account [8,9] than with either a strong automatic attention account [2,3,4] or a simple linguistic nativeness account, under which L1 subtitles would be expected to attract greater engagement [16,17].

One interpretation is that English subtitles (participants’ L2) matched participants’ dominant academic literacy practices more closely than L1 Hindi subtitles. Given participants’ extensive experience with English-medium instruction, English text may have been substantially more practised and familiar in academic contexts than Hindi [15,19,20,21]. Importantly, this does not imply that English was “easier” or preferred for these participants in a global sense, as comprehension scores remained similar across all conditions. Instead, it suggests that English subtitles may have functioned as a more readily usable written channel during instructional viewing than Hindi subtitles, given participants’ script-specific practice background.

In contrast, both L1 Hindi-subtitle conditions elicited reduced subtitle viewing time. In the L1–L1 condition, in which the spoken message was already fully accessible in Hindi, the written channel likely served as a structurally redundant track [12]. According to the Redundancy Principle of multimedia learning [13], processing equivalent auditory and written information simultaneously can impose unnecessary processing demands when one stream is sufficient. The present eye-tracking data suggest that participants may have avoided such redundant processing by shifting their gaze away from the Hindi subtitles. As the auditory signal was fully intelligible, viewers may have had little need to read the subtitles extensively and, instead, allocated attention to the speaker’s face and the visual scene. Importantly, the similar comprehension scores observed across conditions are compatible with this interpretation: reduced subtitle viewing in the L1–L1 condition need not impair comprehension when the written text duplicates information already available through speech [13,14].

More notably, in the L2–L1 condition (English audio, Hindi subtitles), the reduced engagement with subtitles may reflect general multi-channel processing demands associated with cross-linguistic coordination. When viewers are processing academic content in an L2 audio stream, attempting to concurrently decode an L1 text may incur an additional cost with limited utility. As a result, participants may have shifted their attention away from the written channel and focused more on the audiovisual stream, particularly the speaker’s face and speech, which may have provided sufficient support for maintaining comprehension [14].

While our findings are consistent with a literacy utility account, this interpretation is admittedly tentative. Alternative explanations must also be considered. In particular, the lower dwell times on Hindi subtitles may have been modulated by the complexity of the script rather than top-down task utility. Unlike the linear, alphabetic Latin script used for English, the Devanagari script used for Hindi is an alpha-syllabic system featuring non-linear vowel signs, diacritics, and horizontal hanging lines. This layout may increase visual crowding and affect visual word recognition speed [22,23]. Consequently, the reduced gaze duration on Hindi subtitles could reflect the higher visual processing demands for the Devanagari script within this EMI cohort. Future research with Hindi-dominant readers and with EMI bilinguals whose Hindi and English reading fluency is measured directly will be necessary to test this possibility.

Critically, our findings challenge the assumption that subtitles automatically attract attention whenever they are present and legible [2,3,4]. Instead, they reinforce the view that subtitle engagement is dynamic and context-sensitive, reflecting factors like script-specific literacy background and the relative utility of competing informational channels [8,9]. At the same time, the present results do not contradict recent experiments showing the benefits of L1 subtitles in other bilingual groups [16,17]. Instead, they suggest that the usefulness of L1 subtitles is population- and context-dependent. Notably, the 24.4–30.7% dwell time observed in the Hindi-subtitle conditions still indicates that subtitles attracted some visual attention, even though they were not used extensively. However, for bilinguals whose formal literacy practices are strongly tied to an L2, L1 subtitles may not always serve as the primary source of written support during instructional viewing [15].

Several limitations are important when interpreting our findings. First, literacy practice was inferred from the EMI background rather than measured directly through standardised literacy testing. Although this inference is supported by previous sociolinguistic research indicating that EMI students in India may experience reduced formal literacy in their L1 [19,20,21], future work should include objective indices of script-specific reading fluency and reading speed, along with self-reported reading habits in both English and Hindi. Furthermore, given that each participant contributed only one extended video trial per condition, condition-level estimates are less precise than they would be in multi-trial designs with multiple observations per condition. Second, the design did not fully cross the audio and subtitle languages. The Hindi-audio/English-subtitle condition was omitted because it is not representative of the academic and educational exposure of Hindi-native EMI bilinguals. Although this omission increased the ecological relevance of the tested conditions for the target population, we acknowledge that the resulting design prevents a full statistical disentanglement of independent audio-language effects from subtitle-language effects. Third, comprehension questions were presented in English, matching participants’ language of academic instruction. This decision was appropriate for the EMI participants, but it may have encouraged English-mediated processing across conditions. Future research should balance the language of comprehension questions, include comprehension measures less tied to a single language, and test a broader range of instructional materials.

In conclusion, the present findings indicate that subtitle engagement varies with the relation between the written language/script and the viewer’s academic literacy experience. For Hindi–English EMI bilinguals, an L2 written channel in instructional videos may attract more engagement than an L1 written channel when the L2 is the dominant language of formal literacy practice. These results demonstrate that the presence of subtitles alone does not guarantee visual engagement; instead, engagement reflects viewers’ allocation of attention in relation to expected support for comprehension and the processing demands of reading a particular script. In the context of global English-medium instruction, native-language subtitles may not always provide the most readily used written support for academic learning.

## Figures and Tables

**Table 1:** Mean proportional dwell time and standard error (SE) for Face and Subtitle AOIs across the three conditions.

Condition	Face Mean Dwell Time (*SE*)	Subtitles Mean Dwell Time (*SE*)
L2–L2 (English audio + English subtitles)	0.468 (0.052)	0.508 (0.051)
L1–L1 (Hindi audio + Hindi subtitles)	0.676 (0.063)	0.307 (0.061)
L2–L1 (English audio + Hindi subtitles)	0.726 (0.050)	0.244 (0.048)

Note: Face and Subtitle proportions do not sum to 1.00 because gaze spent outside these AOIs contributed to the denominator (total valid sample time).

## Data Availability

The data, scripts, and output files supporting the findings of this study are openly available on the Open Science Framework (OSF) at the following link: https://osf.io/69mys/overview?view_only=285dd5422bb94a148840870a8da7dbae (accessed on 15 April 2026).

## Supplementary Materials

The following supporting information can be downloaded at: https://osf.io/69mys/overview?view_only=285dd5422bb94a148840870a8da7dbae (accessed on 15 April 2026).

## Abbreviations

The following abbreviations are used in this manuscript:

L1	Native language
L2	Second language
EMI	English-medium instruction
AOI	Area of interest
